# Adhesion Potential of Intestinal Microbes Predicted by Physico-Chemical Characterization Methods

**DOI:** 10.1371/journal.pone.0136437

**Published:** 2015-08-21

**Authors:** Tomas de Wouters, Christoph Jans, Tobias Niederberger, Peter Fischer, Patrick Alberto Rühs

**Affiliations:** 1 Laboratory of Food Biotechnology, ETH Zurich, Institute of Food, Nutrition and Health, Schmelzbergstrasse 9, 8092, Zurich, Switzerland; 2 Laboratory of Food Process Engineering, ETH Zurich, Institute of Food, Nutrition and Health, Schmelzbergstrasse 9, 8092, Zurich, Switzerland; LAAS-CNRS, FRANCE

## Abstract

Bacterial adhesion to epithelial surfaces affects retention time in the human gastro-intestinal tract and therefore significantly contributes to interactions between bacteria and their hosts. Bacterial adhesion among other factors is strongly influenced by physico-chemical factors. The accurate quantification of these physico-chemical factors in adhesion is however limited by the available measuring techniques. We evaluated surface charge, interfacial rheology and tensiometry (interfacial tension) as novel approaches to quantify these interactions and evaluated their biological significance via an adhesion assay using intestinal epithelial surface molecules (IESM) for a set of model organisms present in the human gastrointestinal tract. Strain pairs of *Lactobacillus plantarum* WCFS1 with its sortase knockout mutant *Lb*. *plantarum* NZ7114 and *Lb*. *rhamnosus* GG with *Lb*. *rhamnosus* DSM 20021^T^ were used with *Enterococcus faecalis* JH2-2 as control organism. Intra-species comparison revealed significantly higher abilities for *Lb*. *plantarum* WCSF1 and *Lb*. *rhamnosus* GG vs. *Lb*. *plantarum* NZ7114 and *Lb*. *rhamnosus* DSM 20021^T^ to dynamically increase interfacial elasticity (10^−2^ vs. 10^−3^ Pa*m) and reduce interfacial tension (32 vs. 38 mN/m). This further correlated for *Lb*. *plantarum* WCSF1 and *Lb*. *rhamnosus* GG vs. *Lb*. *plantarum* NZ7114 and *Lb*. *rhamnosus* DSM 20021^T^ with the decrease of relative hydrophobicity (80–85% vs. 57–63%), Zeta potential (-2.9 to -4.5 mV vs. -8.0 to -13.8 mV) and higher relative adhesion capacity to IESM (3.0–5.0 vs 1.5–2.2). Highest adhesion to the IESM collagen I and fibronectin was found for *Lb*. *plantarum* WCFS1 (5.0) and *E*. *faecalis* JH2-2 (4.2) whereas *Lb*. *rhamnosus* GG showed highest adhesion to type II mucus (3.8). Significantly reduced adhesion (2 fold) to the tested IESM was observed for *Lb*. *plantarum* NZ7114 and *Lb*. *rhamnosus* DSM 20021^T^ corresponding with lower relative hydrophobicity, Zeta potential and abilities to modify interfacial elasticity and tension. Conclusively, the use of Zeta potential, interfacial elasticity and interfacial tension are proposed as suitable novel descriptive and predictive parameters to study the interactions of intestinal microbes with their hosts.

## Introduction

Increasing awareness of the human body as a supra-organism colonized by a multitude of bacteria has directed research towards the functional role of those seemingly harmless cohabitants. They are found throughout our body and are known to be highly interwoven with human health. The gastro intestinal tract (GI tract) is the most densely colonized body site containing the compelling amount of over 10^11^ bacteria per gram fecal material [1]. This ecosystem is dominated by mainly two phyla, the Firmicutes and Bacteroidetes followed by a much lower amount of Actinobacteria and Proteobacteria. Extensive studies of these bacteria have shown that health effects can often be linked to single species and strains [2]. This specialization of single bacterial species have been observed for immune-stimulation [3–7], metabolic modulations, [8–10] and changes in the metabolite profile of the intestine [11,12]. Therefore laborious screening procedures are used to identify these strains with beneficial effects including extensive phenotypical characterization of candidate strains even within a specific species. The capacity to survive in the GI tract is one of the key characteristics for a beneficial intestinal microbe. To date this capacity is mainly evaluated via characterization of its resistance to acid and bile and adhesion properties to their hosts intestinal epithelial surface molecules (IESM) [13]. Adhesion of bacteria to the IESM increases their retention time and thus their chances to grow and exert beneficial effects in the highly colonized environment of the human GI tract. The first line of contact, the intestinal epithelium is covered by a protective mucus layer exerting a physical barrier function supported by the hydrophobicity of the mucus [14].

Adhesion mechanisms to the gastro-intestinal surface can thus be divided into specific adhesion to IESMs and unspecific adhesion to hydrophobic surfaces in general [15]. Specific adhesion can be mediated by adhesins that physically attach to specific IESM molecules [16–18] or surface attached enzymes such as mucus degrading enzymes that retain specific bacteria in the epithelial mucus layer which covers the intestinal epithelium [19]. Physical proximity of bacteria and their host is required for the initiation of adhesion. Therefore, either the use of active motion to move to the host epithelium [20] or passive movement along physico-chemical gradients that attract bacteria from the intestinal lumen to the hosts epithelial surface are described as means [21,22].

**Fig 1:**
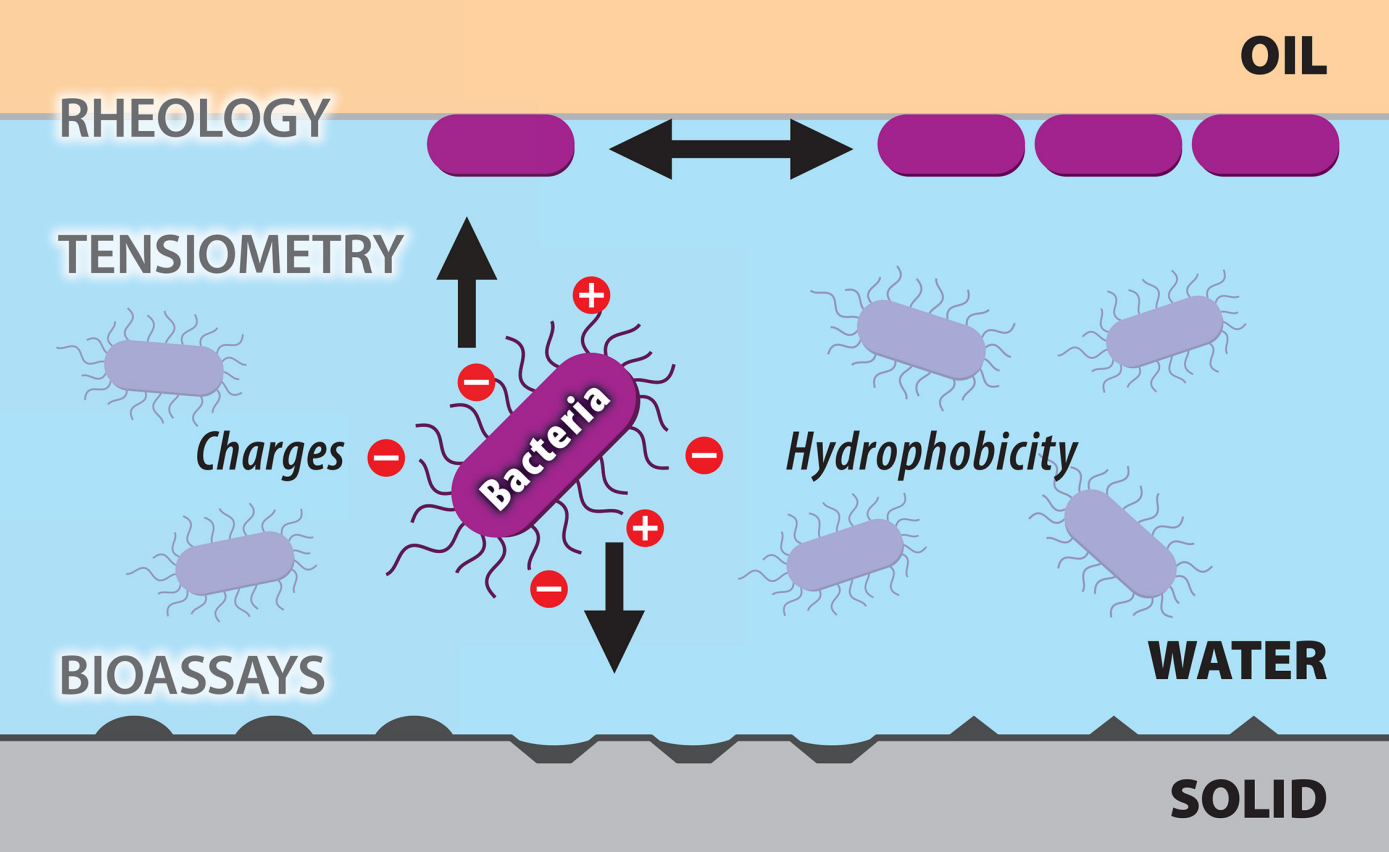
Schematic overview of bacterial adsorption at interfaces. Bacteria are attracted differentially to hydrophobic interfaces depending on their charge density and surface hydrophobicity. These physico-chemical characteristics can be quantified at an oil-water interface as represented in the upper part of the picture. Using rheology interfacial elasticity can be quantified as readout for bacterial adsorption and network development at the interface since they increase interfacial elasticity. Tensiometry quantifies bacterial adsorption through its disruptive effect on the interfacial tension at the oil—water interface. The Zeta potential, a measure of ion strengt can be used as a readoutt for surface charge of bacteria in an aqueous solution and thereby give a quantification of its expected hydrophobicity. The biological significance of these values can be validated *in vitro* through adhesion to specific surface molecules sumarised in the lower part of the picture under bioassays. Thereby the bacteria are applied on a coated surfaces or cell line based models to estimate their adheion potential to different surface molecules represented by symmetric shapes on the lower part of the figure.

In either case physico-chemical interactions are subsequently necessary for the bacterium to attach to the intestinal epithelium. The potential of bacteria to adhere to such hydrophobic interfaces has been studied addressing adhesion to specific surface molecules or intestinal epithelial cells and using indirect methods that characterize general physico-chemical characteristics of candidate bacteria as summarized in Fig 1. The bacterial adhesion to hydrocarbons (BATH) test has widely been used to estimate the physico-chemical component in adhesion capacities of bacterial strains. This indirect method quantifies the surface hydrophobicity of bacteria by quantifying the relative percentage of bacteria retained in a hydrophobic phase after mixing it with an aqueous phase containing the initial bacterial culture. However, the readout of the BATH test is strongly influenced by the solvent used, the experimental pH, and the mixing force applied. The Zeta potential as a measure of ionic charge is a quantifiable variable for the hydrophobicity of both bacterial and hydrocabon surface used in the BATH test [23,24]. The method uses the ability of bacteria to move within an electric field to estimate the bacterial surface charge [25,26]. The Zeta potential is thus an absolute value for bacterial surface charge, which under most physiological conditions has a negative value. Since charge is inversely correlated to hydrophobicity, the absence of a surface charge or a value close to 0 mV will indicate higher affinity of the bacteria to hydrophobic interfaces [27,28]. Thus, based on the hydrophobicity or the charge of the bacterial outer layer, the adhesion ability to hydrophobic surfaces such as mucus can indirectly be characterized.

Recent research established a more elaborated method to precisely quantify bacterial behavior at a hydrophobic interface using interfacial rheology (interfacial viscoelasticity) and tensiometry (interfacial tension) on an oil-water interface completing previously established methods such as BATH test and contact angle measurement [29–32]. These measurements add the possibility of dynamic quantification of interfacial elasticity and interfacial tension upon adsorption of bacteria to an oil-water interface and to consider the interaction between single bacteria upon adsorption to the established methods. The ability of bacteria to adsorb to hydrophobic liquid phases and modify interfacial elasticity was found to be highly strain specific during biofilm formation at water-oil and water-air interfaces [33–37]. However, the biological significance of these indirectly obtained adhesion values require validation in a biologically relevant system. Adhesion assays using either IESM-coated surfaces or intestinal epithelial cell lines provide a relative quantification of bacterial adhesion to different specific molecules on the intestinal surface [38,39]. These methods are thus commonly used to link physico-chemical measurements with their biological relevance.

This study evaluated different physico-chemical methods for the accurate quantification of bacterial adhesion potential in the GI tract and to validate the link between rheological and tensiometric measurements and their biological significance for the prediction of bacterial behavior in the GI tract.

## Material and Methods

### Bacterial strains

*Lactobacillus plantarum* WCFS1 [40] originating from human saliva and *Lactobacillus plantarum* NZ7114, a sortase knockout mutant of *Lb*. *plantarum* WCFS1 [41] were obtained from TI Food and Nutrition (Wageningen, The Netherlands). *Lactobacillus rhamnosus* GG (ATCC 53103) isolated from human feces was obtained from the American Type Culture Collection (ATCC, Manassas, VA, USA). *Lactobacillus rhamnosus* DSM 20021^T^ and *Lactobacillus casei* DSM 20011^T^ (cheese isolate) were obtained from the Deutsche Stammsammlung von Mikroorganismen und Zellkulturen GmbH (DSMZ, Braunschweig, Germany) and used as control strains. *Enterococcus faecalis* JH2-2 [42] was used as fibronectin and collagen I adhesion control. All strains were stored in 30% (v/v) glycerol solution at -80°C.

### Bacterial growth conditions

Brain Heart Infusion broth (BHI; Biolife, Milan, Italy) and MRS broth with Tween 80 (Sigma-Aldrich, Buchs, Switzerland)were used for the general cultivation during adhesion tests of enterococci and lactobacilli, respectively. Growth media were prepared using distilled water and subsequently autoclaved at 121°C for 15 min. All bacteria cultures were subcultured once overnight at 37°C for revitalization from -80°C glycerol stocks.

For all adhesion assays to IESM proteins, strains were inoculated from the first subculture and incubated for 14 hours at 37°C in rubber-sealed screw cap bottles under constant shaking at 160 rpm. The complete biological replication of bacteria cultures for the adhesion assays were performed on three separate days.

For the bacterial characterization and hydrophobicity tests, MRS broth without Tween 80 (Sigma-Aldrich, Buchs, Switzerland) was used to avoid influence of Tween 80 on hydrophobicity measurements [43]. Surfactants in solution would interfere with interfacial tension and elasticity measurements. Therefore for all experiments the strains were inoculated in MRS broth without Tween 80 at 1% (v/v), incubated aerobically at 37°C for 24 hours at 160 rpm resulting in an Tween 80 free stock culture solution that was stored at 4°C. In order to obtain working stock cultures for each experiment the working cultures were inoculated at 1% (v/v) from the stock cultures in MRS broth without Tween 80 and aerobically incubated at 37°C for 24 hours at 160 rpm to obtain active cultures for experimentations.

### Adhesion to extracellular matrix proteins

Fully grown 14-hour bacteria cultures were standardized to an optical density of 1.0 at 600 nm (OD_600_, BIO Photometer, Vaudaux-Eppendorf, Basel, Switzerland) using phosphate-buffered saline (PBS) buffer pH 7.5. PBS was prepared as 10x concentrated stock solution using 40.0 g/LNaCl, 1.0 g/L KCl, 7.2 g/L Na_2_HPO_4_ and 1.2 g/L KH_2_PO_4_ in distilled H_2_O. PBS 1x concentrated was prepared by dilution with distilled water and pH adjustment with 5 M HCl to obtain PBS buffer of pH 7.5 and pH 5.5. All chemicals were obtained from Sigma-Aldrich (Buchs, Switzerland).

Each OD-standardized culture in PBS was then centrifuged at 6,000x g and 20°C for 10 min and the supernatant was carefully poured off. The pellet was resuspended in the original total volume of PBS buffer pH 7.5 and divided into two aliquots of equal volume. Both aliquots were again centrifuged under the same conditions and the supernatant poured off. One aliquot was then resuspended in the same volume of PBS buffer pH 7.5 whereas the other was resuspended in the same volume of PBS buffer pH 5.5 keeping the original OD_600_ = 1.0. Both aliquots of a bacteria strain were stored on ice until further utilization for no longer than 1 h.

All cultures were checked for the absence of bacterial contamination by microscopy and streak-plating onto the corresponding agar medium and incubated under the corresponding growth conditions. For adhesion assays, pure suspensions of single IESMs were used to coat 96-well MaxiSorp plates (Nunc, Roskilde, Denmark). Porcine Type II Mucine (solution 0.5 mg/ml in tris HCl 0.1M pH8, 100 μl/well, Sigma-Aldrich), Collagen I (10 mg/ml in PBS pH 7.5, 100 μl/well, Sigma-Aldrich), Fibrinonectin (10 mg/ml in PBS pH 7.5, 100 μl/well, Sigma-Aldrich) and Fibrinogen (10 mg/ml in PBS pH 7.5, 100 μl/well, Sigma-Aldrich) were used as single IESM proteins and with BSA (0.5 mg/ml in tris HCl 0,1M pH8, Sigma-Aldrich) as unspecific protein adhesion control. Suspensions were applied and left over night at 4°C for adsorption. Liquid was poured off and surface coated plates were dried at 65°C for 10 min. Plates were subsequently blocked with PBS 1% Tween 20 (Sigma-Aldrich) and washed three times with PBS 0.05% Tween 20. Bacterial suspensions (OD_600_ = 1 in PBS, pH 7.5) were applied (100 μl/well), centrifuged at 400 g for 10 min to homogenize contact of bacteria independently of sedimentation speed and incubated for 1 h at 37°C. Non-adhering bacteria were washed off three times with 100 μl PBS 0.05% Tween 20 Sigma-Aldrich, 93773). Adhering bacteria were then fixed for 20 minutes at 65°C and colored (100 μl cristal violet 1 mg/ml in H_2_O, Sigma-Aldrich) for 45 minutes at room temperature. Excess crystal violet was removed with three washing steps using 100 μl of PBS. Remaining crystal violet was solubilized with 100 μl citrate buffer (50Mm, pH 4, Sigma-Aldrich) for 1h at 37°C under constant shaking. The absorption of the resulting solution was measured at a wavelength of 595 nm for maximum absorption of the colorant. The obtained value is directly proportional to the number of adhered bacteria. Experiments were carried out in three biological replicates each comprising three technical replicates. Normalization was performed by multiplying each well with the OD_600_-value of the applied bacterial suspension.

### Physico-chemical interfacial assays

For microscopy, rheology and tensiometry assays, the bacteria were suspended in buffer solutions. As standard buffer, a phosphate buffer comprising sodium phosphate monobasic dihydrate (Sigma-Aldrich) and sodium phosphate dibasic dihydrate (Sigma-Aldrich) at pH 7 and an ionic strength of 100 mM was used. All buffer solutions were made using bidistilled water and autoclaved at 121°C for 15 minutes. As oil phase, mineral oil (Sigma-Aldrich, 330779) was used for all experiments.

For the bacterial adhesion tests, tensiometry, microscopy, determination of the Zeta potential and the BATH-Test, the suspended cells from the measuring cultures, were centrifuged (Biofuge pico, Heraeus and Biofuge primo, Heraeus) and resuspended in buffer solution three times and diluted using buffer solution until OD_600_ = 0.6 was reached (BIO Photometer, Vaudaux-Eppendorf).

### Interfacial rheology

To complement the conventional adhesion methods, interfacial rheology has been introduced as a new technique to study adhesion and network formation of bacteria [35,37]. Rheology is considered the study of the flow of materials, whereas interfacial rheology targets the flow behavior between two immiscible phases such as water-oil or water-air. The aim of interfacial rheology is to determine the mechanical response of adsorption layers by applying shear forces and therefore characterize their viscoelastic properties, reflected by the interfacial moduli [44,45]. As the response to applied shear depends on the composition of the interfacial layer, interfacial rheology yields information on intra- and intermolecular interactions of the probed structure [46–48]. The rheometer measurements were performed on a strain- and stress-controlled rheometer (MCR300, MCR501, and MCR702, Anton Paar, Austria) using a glass measuring cell and a biconical geometry (BIC 68–5, Anton Paar, Austria). Information about the viscoelastic properties was obtained by sinusoidal oscillation of the bicone. In this case, a defined strain γ(t) = γ_0_∙sin(ωt) is applied, which causes a phase shifted stress response τ(t) = τ_0_ sin(ωt + δ), where *δ* is the phase shift. From the strain and stress waves, the complex interfacial shear modulus G_i_* = τ_0_ e^iδ^ ⁄γ_0_ = G_i_^'^ + iG_i_^''^ was calculated. The interfacial storage modulus G_i_^'^ thereby denotes the elastic properties of the interfacial layer and the viscous properties are described by the interfacial loss modulus $G_{i}^{\prime \prime }$. A detailed description of the methodology is presented in the literature [49–51]. Time sweeps were performed at a constant strain and angular frequency (γ = 5% and ω = 0.5 1/s) for 18 h. The temperature was set to 20°C.

### Pendant drop tensiometry

Transient interfacial tension was measured using a pendant drop tensiometer (PAT-1, Sinterface, Germany). A bacteria loaded drop was generated at the tip of a teflon capillary was immersed in mineral oil. The drop area was set to 22 mm^2^ and kept constant over the entire measurement period. During the measurements, the bacteria adhered to the oil-water interface and thereby lowered the interfacial tension. The interfacial tension was then calculated via the the drop shape as described by Ravera and colleagues [51]. The use of the drop tensiometry for surface tension measurement eliminates the third plane in the measurement as compared to alternative methods like the use of a Wilhelmy plate or contact angel measurements [32].

### Bacterial adhesion to hydrocarbons (BATH) test

The BATH test is a method to measure relative hydrophobicity of different bacterial strains [25] which was applied as previously described [35]. In brief: bacteria were washed and diluted to an OD_600_ = 0.6. The cell suspension in PBS buffer at pH 7.0 (100 mM ionic strength) and the mineral oil were mixed at a ratio of 1:0.76 and vortexed for 2 min. After allowing the mixture to rest and separate for 15 minutes, an aliquot of the water phase was taken to measure the OD_600_. To quantify adhesion of the cells to the oil phase, the ratio between the optical density before and after mixing was determined to calculate hydrophobicity according to hydrophobicity in % = (1 -(OD_600_ after mixing) / (OD_600_ before mixing))∙100%

### Electrophoretic mobility

Bacterial cell surface charge was determined using particulate micro-electrophoresis. The electrophoretic mobility μ was calculated by measuring the velocity ʋ_*E*_ of suspended bacteria when an electric field *E* is applied. For a particle with radius *r* and at high ionic strength (κr ≫1) where κ is the Debye length, the Helmholz-Von Smoluchowski equation is valid, which links the electrophoretic mobility to the Zeta potential ζ according to *μ* = ʋ_*E*_/*E* = (ε/η_S_) ζ where *ε* is the dielectric permittivity and *η*_*S*_ is the viscosity of the solvent [26].

### Light microscopy

Microscopy was performed using a Leica DM1000 microscope (Leica Microsystems, Heerbrugg, Switzerland). Washed cells were initially examined at various magnifications using standard microscope slides. Afterwards, the bacteria were studied on hollow cut microscope slides, where the water-oil interface was vertical. Adhesion and network formation was observed over time. The pictures were taken using a Leica DFC280 camera attached to the microscope and the Leica IM50 imaging software.

## Results and Discussion

In order to test the hypothesis of physico-chemical quantifiability of bacterial adhesion properties in the intestinal context, we selected bacterial strains with reported intestinal adhesion properties. We validated their adhesion capacity on an *in vitro* model of the intestinal epithelial cell surface using 96 well plates coated with intestinal epithelial surface molecules and included bovine serum albumin (BSA) coated and untreated wells as controls for unspecific adhesion to non-GI proteins or to the well surface, respectively. We chose *E*. *faecalis* JH2-2 for its well-documented adhesins that bind to collagen-like IESM [52]. *Lb*. *rhamnosus* GG was selected for its specific mucus adhesion properties [17] while *Lb*. *plantarum* WCFS1 was chosen as a well characterized beneficial microbe with unspecific adhesion properties [40]. The cheese isolate *Lb*. *casei* DSM 20011^T^ was used as a *Lactobacillus* without known intestinal adhesion properties.

**Fig 2:**
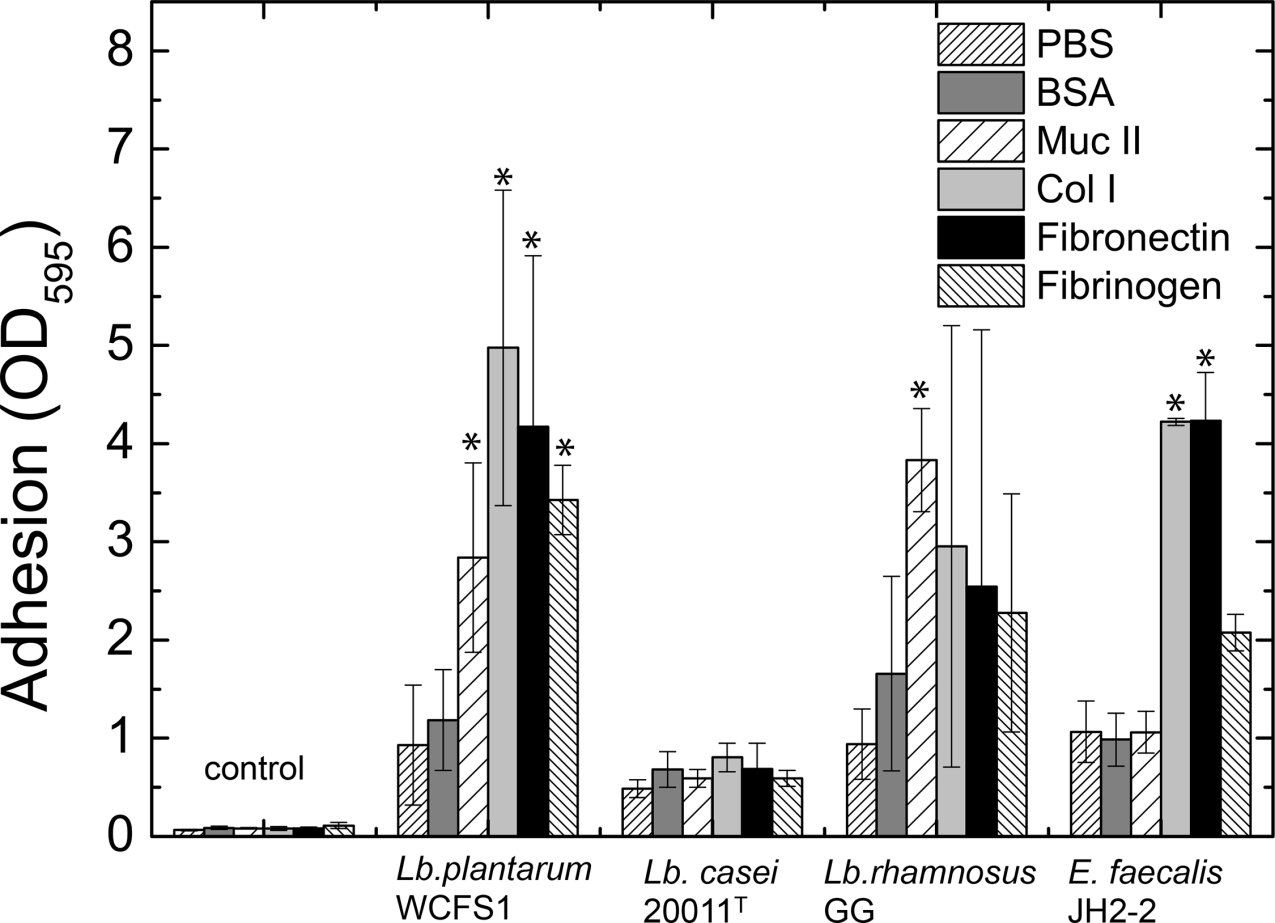
Adhesion properties of reference stains. Adhesion properties to GI tract IESM proteins for the selected reference strains *Lb*. *plantarum* WCFS1, *Lb*. *casei* 20011^T^, *Lb*. *rhamnosus* GG, and *E*. *faecalis* JH2-2. Washed bacteria were exposed for one hour to IESM-coated surfaces in 96-well plates, washed, heat-fixed and colored using crystal violet. Adhesion of bacteria was quantified measuring the absorption of the resolubilized crystal violet at an optical density (OD) of 595 nm that directly correlates with the number of adhering bacteria. Data represent three normalized means of three independent biological replicates each carried out in triplicates. Significance was tested using ANOVA testing with post-hoc Tuckey test. Significance is indicated using * for p < 0.05.

Measured adhesion abilities of GI tract organisms *Lb*. *rhamnosus* GG and *E*. *faecalis* JH2-2 were highest to type II mucin and collagen I as well as fibronectin, respectively (Fig 2) whereas the cheese isolate *Lb*. *casei* DSM 20011^T^ displayed significantly lower adhesion values to nearly all IESMs tested. This confirms the aptitude of the described method for quantification of GI tract-specific or other adhesion properties [17,40,52]. Adhesion to several IESM showed high variances within single tested strains, whereas only the adhesin-mediated adhesion of *Lb*. *rhamnosus* GG to mucus and that of *E*. *faecalis* JH2-2 to collagen I showed high reproducibility and small divergence between experiments. Therefore, statistical significance among the GI tract bacteria could only be calculated for the specific adhesion of *Lb*. *rhamnosus* GG to mucus and *E*. *faecalis* JH2-2 to collagen I and fibronectin. The adhesion profile of *Lb*. *rhamnosus* GG showed no statistical significance for the specific adhesion to collagen I, fibronectin and fibrinogen in the overall analysis despite a visible trend.

In contrast, the oral isolate *Lb*. *plantarum* WCFS1 significantly adhered to all intestinal epithelial surface molecules. However, the adhesion seems to be less specific showing a higher variation between the single biological repetitions. Generally, adhesion profiles are in accordance with the isolation source of each strain with highest mucus adhesion for the GI tract isolate *Lb*. *rhamnosus* GG or fibronectin for the human isolate *E*. *faecalis* JH2-2. In contrast, cheese isolate *Lb*. *casei* DSM 20011^T^ revealed generally low adhesion to the IESM proteins tested.

Related to the hydrophobic properties of mucus, we proceeded to test the hypothesis that the interactions observed for IESM-adherent strains could be based on hydrophobic interactions and thus could be quantified by physico-chemical measurements.

**Fig 3:**
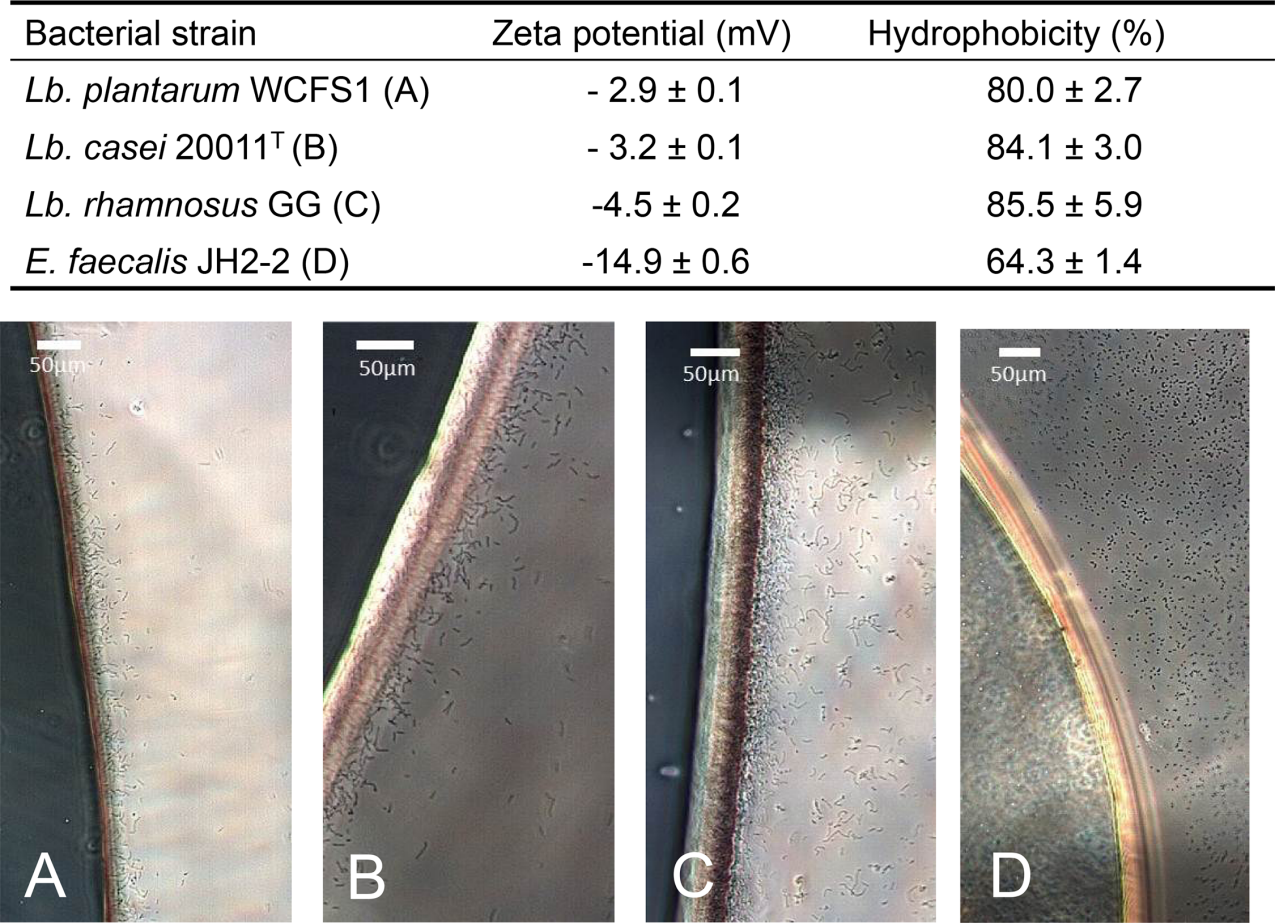
Hydrophobicity and Zeta potential of *Lb*. *plantarum* WCFS1, *Lb*. *casei* 20011^T^, *Lb*. *rhamnosus* GG, and *E*. *faecalis* JH2-2. The Zeta potential was measured through electrophoretic mobility measurements. The hydrophobicity of the bacteria was measured using the BATH test (expressed as % of bacteria soluble in a hydrophobic phase). Microscopic images of *Lb*. *plantarum* WCFS1 (A), *Lb*. *casei* 20011 ^T^ (B), *Lb*. *rhamnosus* GG (C), and *E*. *faecalis* JH2-2 (D) were taken perpendicular to the interface using hollow cut microscopy slides.

Hydrophobicity-based adhesion potential of bacteria was determined by measuring their electrophoretic mobility resulting in the Zeta potential as absolute value and the BATH test as a relative value for hydrophobicity. Generally, decreasing Zeta potential form -2.9 ± 0.1 mV to -14.9 ± 0.6 mV was correlated with a decreasing hydrophobicity from 80.0 ± 2.7% to 64.3 ± 1.4% (Fig 3).

All tested lactobacilli were characterized as strongly hydrophobic bacteria (*Lb*. *plantarum* WCFS1, *Lb*. *casei* 20011^T^ and *Lb*. *rhamnosus* GG), with BATH hydrophobicity values over 80% and Zeta potentials between -2.9 and -4.5 mV as compared to formerly published values of 30% hydrophobicity and -17.3 mV Zeta potential for *Escherichia coli* [35]. *E*. *faecalis* JH2-2, with a hydrophobicity value of 64.3 ± 1.4% and a Zeta potential of -14.9 ± 0.6 mV, was found to be less hydrophobic and more negatively charged in comparison to the other strains. This difference in hydrophobicity and adsorption to hydrophobic interfaces was also observed by microscopy (Fig 3). Lactobacilli showed strong aggregation at the water-oil interface (Fig 3A, 3B and 3C), whereas *E*. *faecalis* JH2-2 (Fig 3D) only weakly adsorbed to the interface.

**Fig 4:**
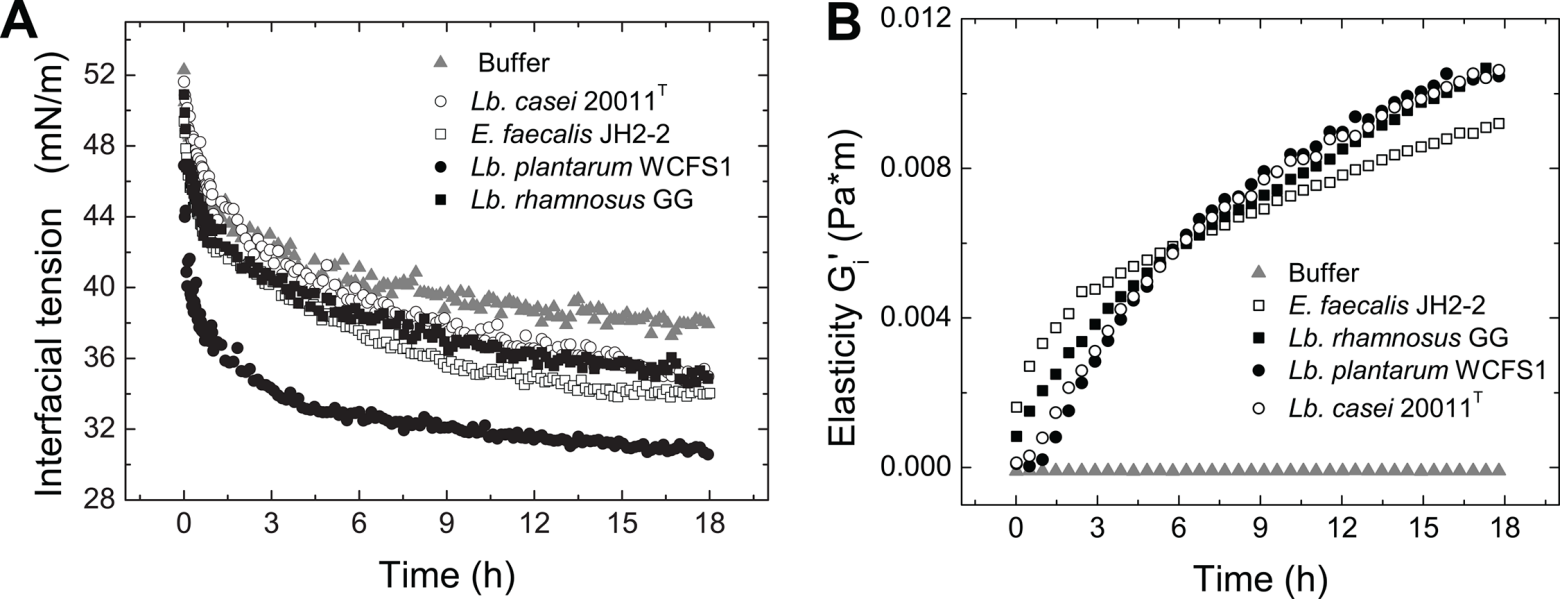
Interfacial rheology and interfacial tension of bacterial adsorption layers at the water-oil interface. A: The interfacial tension decrease over time (0–18 h) was measured with a pendant drop tensiometer. B: The transient elasticity of bacteria in buffer solution was measured with interfacial rheology (0–18 h).

Adsorption of particles, including bacteria, to an interface influences the interfacial tension which can be measured by a rheological and tensiometric approach. Bacterial adsorption at water-oil phases decreased in interfacial tension depending on the adsorbing strain (Fig 4A). The strongest tension decrease was observed for *Lb*. *plantarum* WCFS1 (Fig 4A), which has a Zeta potential closest to 0 mV among the tested strains and a high BATH test hydrophobicity value (Fig 3B). *Lb*. *plantarum* WCFS1 expresses a great number of surface proteins enabling it to adhere to different materials that likely contribute to the overall hydrophobic characteristic and Zeta potential for *Lb*. *plantarum* WCFS1 [40]. Whether these substance serve as biosurfactants as described for *Lactobacillus acidophilus* requires further investigations[53]. However, its high hydrophobicity allows the bacterium to adsorb stronger at the oil phase and thus seems to cause a rapid drop in interfacial tension, possibly influenced by the different surface proteins. In contrast, *Lb*. *casei* 20011^T^, *Lb*. *rhamnosus* GG, and *E*. *faecalis* JH2-2 induced a significantly less pronounced decrease in interfacial tension after 18 hours compared to *Lb*. *plantarum* WCFS1. Even cell-free buffer solution resulted in a change of interfacial tension, which was smaller than all observed changes caused by bacteria and likely due to impurities present in the mineral oil.

The interfacial tension signatures are linked to interfacial rheology of such bacterial adsorption layers at water-air and water-oil interfaces [33–37]. This approach allows quantification of the extent of bacterial adsorption through the increase of interfacial viscoelasticity as a function of the bacterial adsorption layer formation. The resulting mechanical properties are then expressed by the storage modulus G_i_' in its transient evolution (Fig 4B). The loss moduli G_i_'' is not shown, as this value was always lower than G_i_', indicating that these networks were predominantly elastic. All lactobacilli strains formed a highly elastic network of similar strength within 18 hours whereas that of *E*. *faecalis* JH2-2 showed a different time-dependent development (Fig 4B). Plain buffer did not show any increase of elasticity.

The values of the storage modulus reflect directly the bacterial hydrophobicity and their mutual interaction in the adsorption film. Interestingly, *E*. *faecalis* JH2-2 formed a network, that had comparable elastic properties to those formed by *Lb*. *plantarum* WCFS1, *Lb*. *casei* 20011^T^, and *Lb*. *rhamnosus* GG even though its Zeta potential was considerably more negative and thus its hydrophobicity value significantly lower. Despite similar overall characteristics, the curve shape of the development of the network of *E*. *faecalis* JH2-2 is shows a faster incline in the initial 3 h indicated by the strong increase of G_i_', which is then followed by a lower incline during the remaining hours compared to those of the lactobacilli. *E*. *faecalis* JH2-2 is a considerably smaller bacterium, than the *Lactobacilli* tested. This might also explain why *E*. *faecalis* JH2-2 affected the interfacial properties much faster (Fig 4B). *E*. *faecalis* JH2-2 is also known for pillum formation that might increase cell-cell interactions independently of the Zeta potential and physical proximity [54]. In comparison to *E*. *coli* or *B*. *subtilis* featuring significantly higher electronegativity (-17.3 to -31.9 mV) and lower hydrophobicity values (-1.1 to 28.8%) [35], lactobacilli and enterococci tested in the current study still feature considerable hydrophobic properties and thus despite the differences observed between these strains, still exhibit similar tendencies to form elastic networks within 18 hours.

Bacterial membrane properties are largely responsible for hydrophobicity and surface charge. Therefore, they influence adhesion properties and the tendency to form networks, which ultimately influence interfacial rheology or tensiometry. Therefore, the intraspecies strain pairs *Lb*. *plantarum* WCFS1 and *Lb*. *plantarum* NZ7114 as well as *Lb*. *rhamnosus* GG and *Lb*. *rhamnosus* DSM 20021^T^ were evaluated. *Lb*. *plantarum* NZ7114 is a sortase A mutant (*srtA* knock-out) of *Lb*. *plantarum* WCFS1, leading to a significant reduction in surface proteins in *Lb*. *plantarum* NZ7114 [40,41,55].

**Fig 5:**
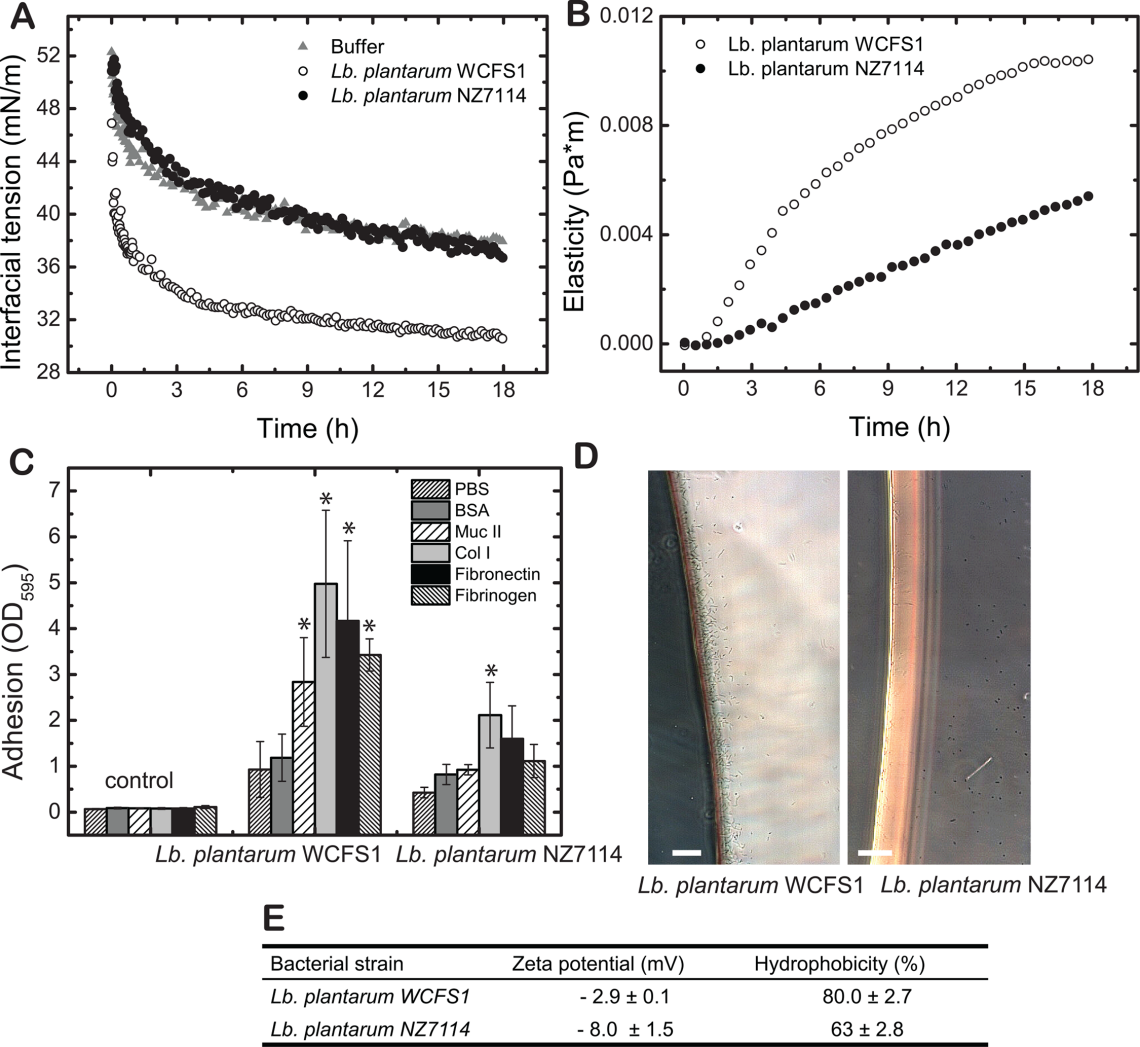
Adhesion characteristics of *Lb*. *plantarum* WCFS1 and NZ7114. A: The transient interfacial tension (A) and elasticity (B) of *Lb*. *plantarum* WCFS1 and NZ7114 are shown for both strains. Adhesion to IESM molecules was measured via the absorption of the re-solubilized crystal violet at OD 595 nm after staining of adhered bacteria, which yields a quantitative value for the number of bacteria adhering. Data represent three normalized means of independent biological replicates each carried out in triplicates. The controls represent coated wells that were not exposed to bacteria, and thus unspecific coloring of crystal violet. Significance was tested using ANOVA testing with post-hoc Tuckey test. Significance is indicated using * for p < 0.05. To reveal if the bacteria adsorb to the oil phase, microscopy images are presented in D. The bacterial properties (Zeta potential and electrophoretic mobility are presented in (E).

The reduced presence of surface proteins *Lb*. *plantarum* NZ7114 decreased the relative hydrophobicity to 63 ± 2.8% and Zeta potential to -8.0 ± 1.5 mV (Fig 5E). Furthermore, these changes in surface properties significantly eliminate the strains capacity to induce interfacial tension decrease yielding similar values as cell-free buffer (Fig 5A) and a reduced effect on the development of interfacial elasticity (Fig 5B). We previously showed that hydrophobicity and thus the capacity to adsorb at the oil-water interface is an important factor for elastic network formation. This network strengthens over time increasing the interfacial elasticity for the wild type strain *Lb*. *plantarum* WCFS1. The *srtA* mutant *Lb*. *plantarum* NZ7114 severely reduced this capacity by the absence of a large fraction of surface proteins, especially LPxTG motif proteins, and consequently reduced hydrophobicity (Fig 5B). *Lb*. *plantarum* NZ7114 also featured a significantly reduced adhesion capability to IESM proteins compared to WCFS1 (Fig 5C) and a decreased adsorption capability at the water-oil interface as observed by microscopy (Fig 5D).

**Fig 6:**
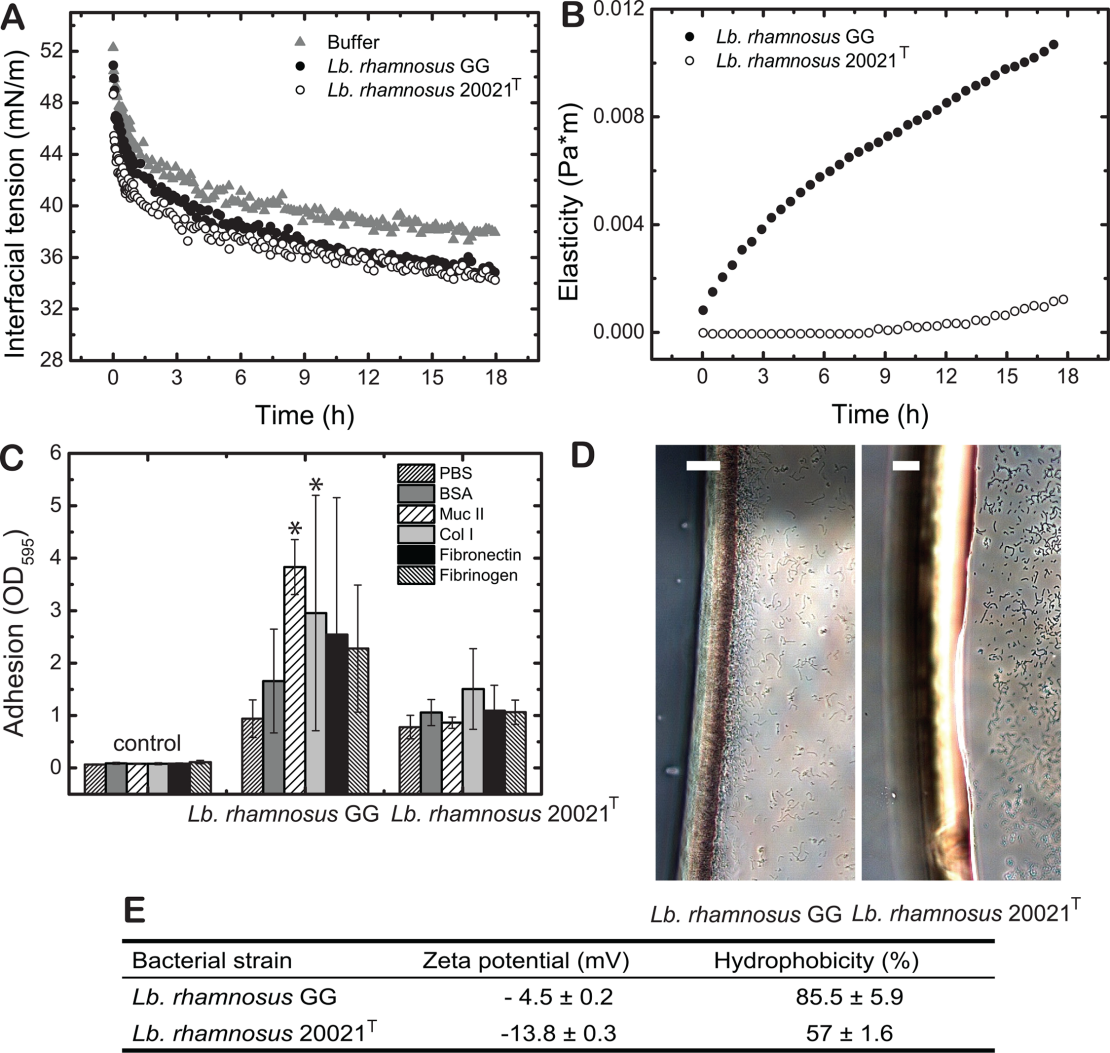
Adhesion characteristics of *Lb*. *rhamnosus* GG and DSM 20021^T^. A: The transient interfacial tension (A) and elasticity (B) of *Lb*. *plantarum* GG and DSM 20021^T^ is shown for both strains. Adhesion to IESM proteins was quantified measuring the absorption of the resolubilized crystal violet at OD 595 nm after staining of adhered bacteria as a quantitative value for the number of bacteria adhering. Data represent three normalized means of independent biological replicates each carried out in triplicates. The controls represent coated wells that were not exposed to bacteria, and thus unspecific coloring of crystal violet. Significance was tested using ANOVA testing with post-hoc Tuckey test. Significance is indicated using * for p < 0.05. To reveal if the bacteria adhere at the oil phase, microscopy images are presented in D. The bacterial properties (Zeta potential and electrophoretic mobility) are presented in the table (E).

A second pair of strains from a single species was used to validate the findings on strain-specific surface properties and their influence on adhesion obtained from *Lb*. *plantarum*. Therefore, the known adhering strain *Lb*. *rhamnosus* GG was compared to the typestrain *Lb*. *rhamnosus* DSM 20021^T^ (Fig 6) for which no adhesion properties are described. Zeta potential and relative hydrophobicity were significantly lower for *Lb*. *rhamnosus* DSM 20021^T^ compared to *Lb*. *rhamnosus* GG (Fig 6E). Both strains lowered the interfacial tension to the same extent (Fig 6A), however the more hydrophobic strain *Lb*. *rhamnosus* GG clearly increased interfacial elasticity while *Lb*. *rhamnosus* DSM 20021^T^ showed only marginal effect (Fig 6B). The ability of *Lb*. *rhamnosus* DSM 20021^T^ to attach to IESM proteins and especially mucus or collagen I was significantly lower in contrast to strain GG (Fig 6C) in correlation also with lower adsorption capacities at the water-oil interface (Fig 6D) and lower IESM protein-specific adhesion.

Therefore, the diminution of surface proteins, mainly sortase-dependent of LPxTG motif, in the *srtA* mutant *Lb*. *plantarum* NZ7114 strain and the potential surface differences between *Lb*. *rhamnosus* GG and DSM 20021^T^ suggest a strong influence of these proteins in overall cell hydrophobicity, electric charge and eventually adhesion properties, which is quantifiable by interfacial tensiometry and elasticity.

## Conclusion

**Fig 7:**
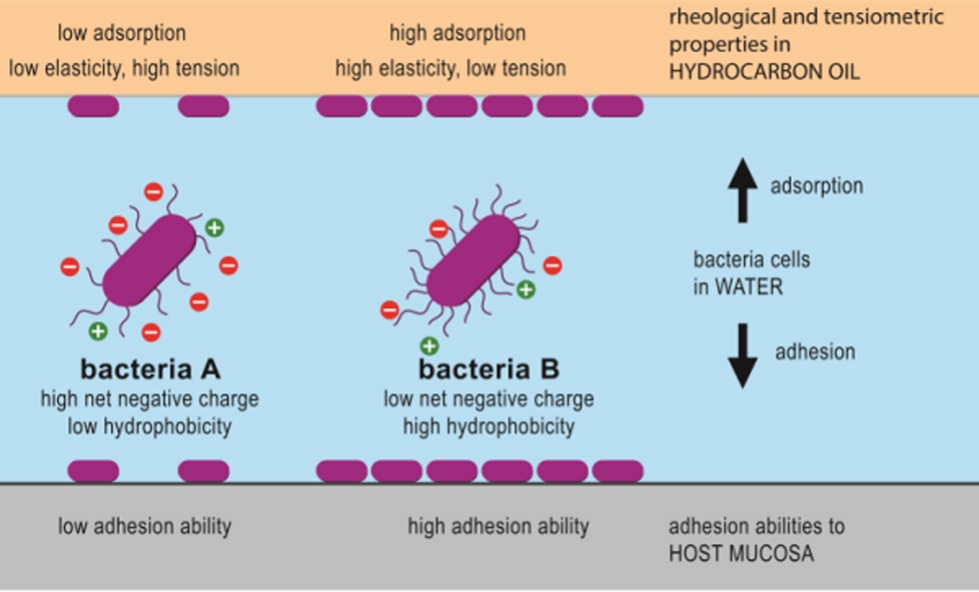
Summary of bacterial adhesion. The capacity of bacteria to adhere is a function of physico-chemical charges and surface properties of a bacteria. These properties can be measured quantitatively using rheological and tensiometric methods as illustrated in the upper part of the figure. Through bacterial adsorption at a hydrophobic interface, the interfacial elasticity is increased and depending on the bacterial characteristics, interfacial tension can be decreased. These measurable parameters can be used as quantitative measures for physico-chemical characteristics to different bacterial strains. These physicochemical properties can be used to predict bacteria's potential to adhere to biological surfaces like the intestinal mucosa as illustrated in the lower part of the figure.

Intestinal bacteria are under constant competition for nutrients and colonization of their niche. Increased retention in the intestine through adhesion to the intestinal epithelium is an important competitive advantage for many beneficial microbes. The novel measurements via interfacial rheology, interfacial viscoelasticity, and interfacial tension of these properties can be utilized as quantifiable predictors of adhesion capacity of a strain, allowing an estimation of bacterial adsorption kinetics and bacterial layer formation in physiological conditions (Fig 7). Therefore we present Zeta potential, interfacial viscoelasticity and interfacial tension as quantitative, reproducible, and comparable measures to characterize the surface properties of intestinal bacteria, and propose these rheological measures as important descriptive and predictive parameters in the study of intestinal microbes colonizing the intestinal epithelium.

## References

[1] Ley RE, Hamady M, Lozupone CA, Turnbaugh PJ, Ramey RR, Bircher JS, et al Evolution of mammals and their gut microbes. Science. 2008;777: 1647–1651. 10.1126/science.1155725 PMC2649005 18497261

[2] Bron PA, Tomita S, Mercenier A, Kleerebezem M. Cell surface-associated compounds of probiotic lactobacilli sustain the strain-specificity dogma. Curr Opin Microbiol. 2013;16: 262–9. 10.1016/j.mib.2013.06.001 23810459

[3] Ivanov II, Atarashi K, Manel N, Brodie EL, Shima T, Karaoz U, et al Induction of Intestinal Th17 Cells by Segmented Filamentous Bacteria. Cell. 2009;139: 485–498. 10.1016/j.cell.2009.09.033 19836068 PMC2796826

[4] Fukuda S, Toh H, Hase K, Oshima K, Nakanishi Y, Yoshimura K, et al Bifidobacteria can protect from enteropathogenic infection through production of acetate. Nature. Nature Publishing Group; 2011;469: 543–547. 10.1038/nature09646 21270894

[5] Round JL, Mazmanian SK. Inducible Foxp3+ regulatory T-cell development by a commensal bacterium of the intestinal microbiota. Proc Natl Acad Sci U S A. 2010;107: 12204–9. 10.1073/pnas.0909122107 20566854 PMC2901479

[6] Mazmanian SK, Round JL, Kasper DL. A microbial symbiosis factor prevents intestinal inflammatory disease. Nature. 2008;453: 620–5. 10.1038/nature07008 18509436

[7] Lakhdari O, Tap J, Béguet-Crespel F, Le Roux K, de Wouters T, Cultrone A, et al Identification of NF-κB Modulation Capabilities within Human Intestinal Commensal Bacteria. J Biomed Biotechnol. 2011;2011: 282356 10.1155/2011/282356 21765633 PMC3134244

[8] Valladares R, Sankar D, Li N, Williams E, Lai K-K, Abdelgeliel AS, et al *Lactobacillus johnsonii* N6.2 mitigates the development of type 1 diabetes in BB-DP rats. Stadler K, editor. PLoS One. Public Library of Science; 2010;5: e10507 10.1371/journal.pone.0010507 20463897 PMC2865539

[9] Everard A, Belzer C, Geurts L, Ouwerkerk JP, Druart C, Bindels LB, et al Cross-talk between *Akkermansia muciniphila* and intestinal epithelium controls diet-induced obesity. Proc Natl Acad Sci U S A. 2013;110: 9066–71. 10.1073/pnas.1219451110 23671105 PMC3670398

[10] Cotillard A, Kennedy SP, Kong LC, Prifti E, Pons N, Le Chatelier E, et al Dietary intervention impact on gut microbial gene richness. Nature. 2013;500: 585–8. 10.1038/nature12480 23985875

[11] Musso G, Gambino R, Cassader M. Interactions between gut microbiota and host metabolism predisposing to obesity and diabetes. Annu Rev Med. 2011;62: 361–80. 10.1146/annurev-med-012510-175505 21226616

[12] Brestoff JR, Artis D. Commensal bacteria at the interface of host metabolism and the immune system. Nat Immunol. 2013;14: 676–684. 10.1038/ni.2640 23778795 PMC4013146

[13] Fontana L, Bermudez-Brito M, Plaza-Diaz J, Muñoz-Quezada S, Gil A. Sources, isolation, characterisation and evaluation of probiotics. Br J Nutr. 2013;109 Suppl: S35–50. 10.1017/S0007114512004011 23360880

[14] Lichtenberger LM. The Hydrophobic Barrier Properties of Gastrointestinal Mucus. Annu Rev Physiol. 1995;57: 565–583. 7778878 10.1146/annurev.ph.57.030195.003025

[15] Poortinga A, Bos R, Norde W. Electric double layer interactions in bacterial adhesion to surfaces. 2002;47: 3–32.

[16] Nallapareddy SR, Qin X, Weinstock GM, Höök M, Murray BE. *Enterococcus faecalis* adhesin, ace, mediates attachment to extracellular matrix proteins collagen type IV and laminin as well as collagen type I. Infect Immun. 2000;68: 5218–24. 10948147 10.1128/iai.68.9.5218-5224.2000 PMC101781

[17] Kankainen M, Paulin L, Tynkkynen S, von Ossowski I, Reunanen J, Partanen P, et al Comparative genomic analysis of *Lactobacillus rhamnosus* GG reveals pili containing a human- mucus binding protein. Proc Natl Acad Sci U S A. 2009;106: 17193–8. 10.1073/pnas.0908876106 19805152 PMC2746127

[18] Pamp SJ, Harrington ED, Quake SR, Relman DA, Blainey PC. Single-cell sequencing provides clues about the host interactions of segmented filamentous bacteria (SFB). Genome Res. 2012;22: 1107–19. 10.1101/gr.131482.111 22434425 PMC3371716

[19] Belzer C, de Vos WM. Microbes inside—from diversity to function: the case of *Akkermansia* . ISME J. 2012;6: 1449–58. 10.1038/ismej.2012.6 22437156 PMC3401025

[20] Marteyn B, West NP, Browning DF, Cole J a, Shaw JG, Palm F, et al Modulation of *Shigella* virulence in response to available oxygen in vivo. Nature. 2010;465: 355–8. 10.1038/nature08970 20436458 PMC3750455

[21] Xu H, Jeong HS, Lee HY, Ahn J. Assessment of cell surface properties and adhesion potential of selected probiotic strains. Lett Appl Microbiol. 2009;49: 434–42. 10.1111/j.1472-765X.2009.02684.x 19725886

[22] Pan Y, F. BJ, Kathariou S. Resistance of Listeria monocytogenes biofilms to sanitizing agents in a simulated food processing environment. Appl Environ Microbiol. 2006;72: 7711–7717. 17012587 10.1128/AEM.01065-06 PMC1694257

[23] Busscher HJ, van de Belt-Gritter B, van der Mei HC. Implications of microbial adhesion to hydrocarbons for evaluating cell surface hydrophobicity 1. Zeta potentials of hydrocarbon droplets. Colloids Surfaces B Biointerfaces. 1995;5: 111–116. 10.1016/0927-7765(95)01224-7

[24] Van der Mei HC, van de Belt-Gritter B, Busscher HJ. Implications of microbial adhesion to hydrocarbons for evaluating cell surface hydrophobicity 2. Adhesion mechanisms. Colloids Surfaces B Biointerfaces. 5: 117–126. 10.1016/0927-7765(95)01225-8

[25] Rosenberg M, Gutnick D, Rosenberg E. Adherence of bacteria to hydrocarbons: A simple method for measuring cell-surface hydrophobicity. FEMS Microbiol Lett. 1980;9: 29–33. 10.1111/j.1574-6968.1980.tb05599.x

[26] Poortinga AT. Electric double layer interactions in bacterial adhesion to surfaces. Surf Sci Rep. Elsevier; 2002;47: 1–32.

[27] Van Loosdrecht MC, Lyklema J, Norde W, Schraa G, Zehnder AJ. The role of bacterial cell wall hydrophobicity in adhesion. Appl Environ Microbiol. 1987;53: 1893–1897. 2444158 10.1128/aem.53.8.1893-1897.1987 PMC204020

[28] Van Loosdrecht MC, Lyklema J, Norde W, Schraa G, Zehnder AJ. Electrophoretic mobility and hydrophobicity as a measured to predict the initial steps of bacterial adhesion. Appl Environ Microbiol. 1987;53: 1898–901. 3662520 10.1128/aem.53.8.1898-1901.1987 PMC204021

[29] Bussher H, Weerkamp A, Vandermei H, Vanpelt A, Dejong H, Arends J. Measurement of the Surface Free Energy of Bacterial Cell Surfaces and Its Relevance for Adhesion. Appl Environ Microbiol. 1984;48: 980–983. 6508312 10.1128/aem.48.5.980-983.1984 PMC241661

[30] Busscher H., Van Pelt a. W., De Jong H., Arends J. Effect of spreading pressure on surface free energy determinations by means of contact angle measurements. J Colloid Interface Sci. 1983;95: 23–27. 10.1016/0021-9797(83)90067-X

[31] Busscher HJ, van Pelt AWJ, de Boer P, de Jong HP, Arends J. The effect of surface roughening of polymers on measured contact angles of liquids. Colloids and Surfaces. 1984;9: 319–331. 10.1016/0166-6622(84)80175-4

[32] Van der Mei HC, Weerkamp a. H, Busscher HJ. A comparison of various methods to determine hydrophobic properties of streptococcal cell surfaces. J Microbiol Methods. 1987;6: 277–287. 10.1016/0167-7012(87)90065-0

[33] Wu C, Lim JY, Fuller GG, Cegelski L. Quantitative Analysis of Amyloid-Integrated Biofilms Formed by Uropathogenic Escherichia coli at the Air-Liquid Interface. Biophys J. 2012;103: 464–471. 10.1016/j.bpj.2012.06.049 22947862 PMC3414876

[34] Wu C, Lim JY, Fuller GG, Cegelski L. Disruption of Escherichia coli Amyloid-Integrated Biofilm Formation at the Air-Liquid Interface by a Polysorbate Surfactant. Langmuir. 2013;29: 920–926. 10.1021/la304710k 23259693 PMC3557966

[35] Rühs PA, Böcker L, Inglis RF, Fischer P. Studying bacterial hydrophobicity and biofilm formation at liquid-liquid interfaces through interfacial rheology and pendant drop tensiometry. Colloids Surf B Biointerfaces. 2014;117: 174–84. 10.1016/j.colsurfb.2014.02.023 24632390

[36] Hollenbeck EC, Fong JCN, Lim JY, Yildiz FH, Fuller GG, Cegelski L. Molecular Determinants of Mechanical Properties of V. cholerae Biofilms at the Air-Liquid Interface. Biophys J. Elsevier; 2014;107: 2245–2252. 10.1016/j.bpj.2014.10.015 25418293 PMC4241461

[37] Rühs PA, Böni L, Fuller GG, Inglis RF, Fischer P. In-situ quantification of the interfacial rheological response of bacterial biofilms to environmental stimuli. PLoS One. 2013;8: e78524 10.1371/journal.pone.0078524 24244319 PMC3823922

[38] Stepanović S, Ćirković I, Ranin L, Suivabić-Vlahović M. Biofilm formation by *Salmonella* spp. and *Listeria monocytogenes* on plastic surface. Lett Appl Microbiol. Blackwell Science Ltd; 2004;38: 428–432. 10.1111/j.1472-765X.2004.01513.x 15059216

[39] Servin AL, Coconnier M-H. Adhesion of probiotic strains to the intestinal mucosa and interaction with pathogens. Best Pract Res Clin Gastroenterol. 2003;17: 741–754. 10.1016/S1521-6918(03)00052-0 14507585

[40] Kleerebezem M, Boekhorst J, van Kranenburg R, Molenaar D, Kuipers OP, Leer R, et al Complete genome sequence of *Lactobacillus plantarum* WCFS1. Proc Natl Acad Sci U S A. 2003;100: 1990–5. 10.1073/pnas.0337704100 12566566 PMC149946

[41] Remus DM, Bongers RS, Meijerink M, Fusetti F, Poolman B, de Vos P, et al Impact of *Lactobacillus plantarum* sortase on target protein sorting, gastrointestinal persistence, and host immune response modulation. J Bacteriol. 2013;195: 502–9. 10.1128/JB.01321-12 23175652 PMC3554011

[42] Jacob AE, Douglas GJ, Hobbs SJ. Self-Transferable Plasmids Determining the Hemolysin and Bacteriocin of *Streptococcus faecalis* var. zymogenes. J Bacteriol. 1975;121: 863–872. 803965 10.1128/jb.121.3.863-872.1975 PMC246013

[43] De Man JC, Rogosa M, Sharpe ME. A medium for the cultivation of Lactobacilli. J appl Bact. 1960;23 (1): 130–135.

[44] Sagis LMC. Dynamic properties of interfaces in soft matter: Experiments and theory. Rev Mod Phys. 2011;83: 1367–1403.

[45] Erni P. Deformation modes of complex fluid interfaces. Soft Matter. The Royal Society of Chemistry; 2011;7: 7586–7600. 10.1039/C1SM05263B

[46] Miller R, Ferri J, Javadi A, Krägel J, Mucic N, Wüstneck R. Rheology of interfacial layers. Colloid Polym Sci. Springer-Verlag; 2010;288: 937–950. 10.1007/s00396-010-2227-5

[47] Krägel J, Derkatch SR. Interfacial shear rheology. Curr Opin Colloid Interface Sci. 2010;15: 246–255. 10.1016/j.cis.2008.08.010 18823871

[48] Sagis LMC, Fischer P. Nonlinear rheology of complex fluid–fluid interfaces. Curr Opin Colloid Interface Sci. 2014;19: 520–529. 10.1016/j.cocis.2014.09.003

[49] Erni P, Fischer P, Windhab EJ, Kusnezov V, Stettin H, Läuger J. Stress- and strain-controlled measurements of interfacial shear viscosity and viscoelasticity at liquid/liquid and gas/liquid interfaces. Rev Sci Instrum. 2003;74: 4916–4924.

[50] Rühs PA, Scheuble N, Windhab EJ, Mezzenga R, Fischer P. Simultaneous Control of pH and Ionic Strength during Interfacial Rheology of beta-Lactoglobulin Fibrils Adsorbed at Liquid/Liquid Interfaces. Langmuir. 2012;28: 12536–12543. 10.1021/la3026705 22857147

[51] Ravera F, Loglio G, Kovalchuk VI. Interfacial dilational rheology by oscillating bubble/drop methods. Curr Opin Colloid Interface Sci. 2010;15: 217–228.

[52] Jacob A. E., Hobbs S. J. Conjugal transfer of plasmid-borne multiple antibiotic resistance in *Streptococcus faecalis* var. *zymogenes* . J Bacteriol. 1974;117: 360–372. 4204433 10.1128/jb.117.2.360-372.1974 PMC285522

[53] Velraeds MM, van der Mei HC, Reid G, Busscher HJ. Inhibition of initial adhesion of uropathogenic *Enterococcus faecalis* to solid substrata by an adsorbed biosurfactant layer from *Lactobacillus acidophilus* . Urology. 1997;49: 790–4. 10.1016/S0090-4295(97)00065-4 9145994

[54] Kline KA, Kau AL, Chen SL, Lim A, Pinkner JS, Rosch J, et al Mechanism for sortase localization and the role of sortase localization in efficient pilus assembly in *Enterococcus faecalis* . J Bacteriol. 2009;191: 3237–47. 10.1128/JB.01837-08 19286802 PMC2687161

[55] Boekhorst J, Wels M, Kleerebezem M, Siezen RJ. The predicted secretome of *Lactobacillus plantarum* WCFS1 sheds light on interactions with its environment. Microbiology. 2006;152: 3175–83. 10.1099/mic.0.29217-0 17074889

